# Association of Lp-PLA_2_ Mass and Aysmptomatic Intracranial and Extracranial Arterial Stenosis in Hypertension Patients

**DOI:** 10.1371/journal.pone.0130473

**Published:** 2015-06-22

**Authors:** Yan Wang, Jin Zhang, Yuesheng Qian, Xiaofeng Tang, Huawei Ling, Kemin Chen, Pingjin Gao, Dingliang Zhu

**Affiliations:** 1 Research Center for Hypertension Management and Prevention in Community, Shanghai Key Laboratory of Hypertension, Shanghai Institute of Hypertension, State Key Laboratory of Medical Genomics, Ruijin Hospital, Shanghai Jiaotong University School of Medicine, Shanghai, China; 2 Department of Radiology, Ruijin Hospital, Shanghai Jiaotong University School of Medicine, Shanghai, China; Massachusetts General Hospital, UNITED STATES

## Abstract

**Background and Purpose:**

Intracranial arterial stenosis (ICAS) is a common cause of ischemic stroke in Asians, whereas whites tend to have more extracranial lesions. Lipoprotein-associated phospholipase A_2_ (Lp-PLA_2_) has been associated with ischemic stroke by a large amount of work. However, there are few studies focusing on the relationship of Lp-PLA_2_ and asymptomatic ICAS or extracranial arterial stenosis (ECAS). Wehereby sought to explore the relationship of Lp-PLA_2_ and ICAS, ECAS and concurrent stenosis in stroke-free hypertensive patients in Chinese population.

**Methods:**

All the subjects were evaluated for the presence and severity of ICAS and ECAS through computerized tomographic angiography (CTA) covered the whole brain down to the level of aortic arch. Lp-PLA_2_ mass was measured by enzyme linked immunoassay. The association of Lp-PLA_2_ and vascular stenosis was analyzed through multivariate logistic regression.

**Results:**

Among 414 participants, 163 (39.4%) had no ICAS or ECAS, 63 (15.2%) had ECAS only, 111 (26.8%) had ICAS only and 77 (18.6%) had concurrent extraintracranial stenosis. Lp-PLA_2_ mass was significantly associated with isolated ICAS (OR: 2.3; 95% CI: 1.14-4.64), and concurrent stenosis (OR: 3.93; 95% CI: 1.62-9.51), but was not related to isolated ECAS (OR: 1.54; 95% CI: 0.68-3.48). Lp-PLA_2_ mass was also associated with moderate to severe ICAS no matter how was the ECAS. Moreover, patients with higher Lp-PLA_2_ mass showed more sever ICAS and had more intracranial arterial lesions.

**Conclusion:**

This study revealed the association of Lp-PLA_2_ mass with ICAS in stroke-free hypertensive patients in Chinese population. The further long-term cohort study was warranted to elucidate the concrete effect of Lp-PLA_2_ on the asymptomatic ICAS.

## Introduction

Stroke is a global health problem and is the leading cause of death in China [1], with ischemic stroke as the predominant subtype. Intracranial arterial stenosis (ICAS) is a more common cause of ischemic stroke in Asians, whereas whites tend to have more extracranial lesions [2–4]. Meanwhile, hypertension is a well-established risk factor for both ischemic and hemorrhagic forms of stroke, as well as for ICAS [2, 5, 6]. However, there is limited study on the risk factors of asymptomatic ICAS, extracranial arterial stenosis (ECAS) and concurrent stenosis in hypertension patients.

Lipoprotein-associated phospholipase A_2_ (Lp-PLA_2_) can cleave the oxidized fatty acid side chain at the sn2 position of oxidized phospholipids, which generates lysophosphatidylcholine and oxidized free fatty acid，and mediates multiple inflammatory pathway [7, 8]. Recently, elevated circulating levels of Lp-PLA_2_ have been shown to be independent predictors of coronary heart disease and ischemic stroke in general population and clinical patients [9, 10]. However, there is no study focusing on the relationship of Lp-PLA_2_ and asymptomatic ICAS or ECAS. Therefore, in the present study, we aim to investigate the association of Lp-PLA_2_ and ICAS, ECAS and concurrent stenosis in stroke-free hypertensive patients in Chinese population.

## Methods

### Study design

This study was undertaken within the framework of an ongoing cross-section and prospective study in China, which was a computerized tomographic angiography (CTA) based study of intra- and extracranial asymptomatic artery stenosis and stroke outcome in stroke-free hypertension patients. Subjects of this study were recruited from hypertension outpatients who were identified in Xinzhuang Community hospital between May 2012 and May 2013, and then referred to Ruijin Hospital, a general hospital in Shanghai. Hypertensive status was defined as systolic BP (SBP) ≥ 140mmHg and/ordiastolic BP (DBP) ≥ 90mmHg, or individuals taking antihypertensive medication. All the participants were over 50 years old. Those who had stroke, transient ischemic attack (TIA), or atrial fibrillation identified from medical history was excluded. Those who were unfit for CTA examination because of iodine allergy were also excluded.

### Ethics statement

The study protocol was approved by the ethics committee of Ruijin Hospital and written informed consent was obtained from all participants.

### Demographic and clinical measurements

After the subjects had rested for at least 5 min in the sitting position, SBP and DBP were measured using a verified electronic sphygmomanometer (OMRON, HEM-907) by a trained physician or nurse. The average of three consecutive BP readings with one minute interval of each participant was used for the current analysis. Body weight and height were recorded with participants wearing light indoor clothing and no shoes. Clinical information was collected by interview, including smoking and drinking habits, current drug intake, personal and family history of hypertension and diabetes, etc. Current smokers were defined as those who had smoked cigarettes on one or more days in the past 30 days. All the biochemical measurements including fasting plasma glucose, serum concentrations of total cholesterol (TC), triglycerides (TGs), high-density lipoprotein cholesterol (HDL), low-density lipoprotein cholesterol (LDL), serum creatinine, serum urea nitrogen, uric acid, urine albumin creatinine ratio and neutrophil accounts were performed in the Central Laboratory of Ruijin Hospital (Shanghai, China) using the standard protocols.

Lp-PLA_2_ mass was measured in plasma aliquots stored in -80°C freezers, using an enzyme linked immunoassay. Samples were incubated in microtitre plate wells with immobilized monoclonal antibody against Lp-PLA_2_ (R&D AF5106,MN，USA), and then it was identified by an anti-Lp-PLA_2_ antibody labeled with horseradish peroxidase. The range of detection was 40 to 1200ng/ml. Lp-PLA_2_ mass was measured in duplicate with intra-assay CV of 2.6%.

### CTA protocol

CTA was performed with a 64-section helical CT scanner (GE FX/I, General Electric, Fairfield, CT) as our previous prescription [11]. CTA acquisitions were obtained after a single bolus intravenous injection of 70 ml OptirayIoversol 320 into the antecubital vein at a rate of 3 ml/ sec. Scanning covered the whole brain down to the level of aortic arch with 5-mm slice thickness. Images were reformatted in axial, sagittal, and coronal planes with 1.25-mm slice thickness. All images were read at a workstation with the software of AW4.4 vessel analysis independently by two experienced radiologist who were blinded to clinical data of the patients. Stenosis was defined as a lesion that decreased arterial internal diameter. The percentage of stenosis was calculated as the ratio of the diameter of the diseased artery at its most severe site divided by the diameter of a nearby normal segment. The degree of stenosis was categorized into mild (<30%), moderate (30–69%), or severe (> = 70%). The intracranial arteries included intracranial segment of internal carotid artery and vertebral artery, basilar artery, anterior cerebral artery, middle cerebral artery and posterior cerebral artery. The greatest stenosis at an intracranial or extracranial artery was chosen as being representative for each subject. The extracranial arteries included extracranial segment of internal carotid artery and vertebral artery, external carotid artery, common carotid artery and subclavian artery. The number of arteries with stenosis for each patient was also counted. The two radiologists had good agreement in the designation of stenosis (κ = 0.93, P < 0.001). All disagreements were reviewed and adjudicated by a senior radiologist to reach a consensus.

### Statistical analysis

For database management and statistical analysis, we used SPSS software (version 13.0; SPSS Inc., Chicago, Illinois, USA). Descriptive statistics for patients with or without ICAS and ECAS were compared using a Pearson Chi-square test for categorical variables and Student t test for continuous variables. Correlation was evaluated by Spearman coefficients. Multivariate logistic regression was performed to test the association of Lp-PLA_2_ mass with risk of ICAS and ECAS separately in two models. The basal model was adjusted for sex and age. Further analysis was adjusted for BMI, hypertension duration, current smoking and drinking status, diabetes, LDL, HDL, plasma glucose, mean arterial pressure, heart rate, neutrophil account, urine albumin creatinine ratio, serum creatinine, anti-hypertensive treatment and statin use in addition. Logarithmic transformation was used on variables that were not normally distributed. All *P* values were 2-tailed, and a *P* value of <0.05 was considered statistically signiﬁcant.

## Results

The general characteristics of patients according to the location and severity of stenosis were shown in Table 1. Of 414 subjects included in the study, 39.4% had no ICAS or ECAS, 15.2% had ECAS only, 26.8% had ICAS only, and 18.6% had concurrent extraintracranial artery stenosis. Among the 67 subjects with moderate to severe stenosis, 60.3% had ECAS only, 31.5% had ICAS only, and 18.2% had concurrent stenosis. Comparing with the stenosis absent group, the patients with ICAS were older and with higher frequency of male, longer hypertension duration, higher SBP, slower heart rates, higher serum creatinine and Lp-PLA_2_ mass. And the patients with moderate to severe ICAS were more likely to be smoking, with the comorbidity of diabetes and lower HDL.

**Table 1:** Clinical characteristics of hypertensive patients according to location and severity of arterial stenosis.

Baseline characteristics	Control	Mild to severe stenosis			Moderate to severe stenosis		
	ICAS/ECAS absent	ECAS present	ICAS present	COMB present	ECAS present	ICAS present	COMB present
N	163	63	111	77	38	35	14
Age (years)	64.4±5.9	65.5±5.5	67.1±5.6†	68.3±5.4†	67.1±5.8*	66.7±5.7*	68.9±5.6†
Male (N,%)	60(36.8)	32(50.8)	60(54.1)†	44(57.1)†	13 (34.2）	15 (42.90）	5(57.1）
Smoking (N,%)	17(10.4)	12(19)	20(18)	16(20.8)	9(23.7)*	9(25.7)*	2(14.3)
Dringking (N,%)	24(14.7)	9(14.3)	18(16.2)	10(13)	6(15.8)	5(14.3)	2(14.3)
Diabetes mellitus (N,%)	38 (23.3)	9 (14.3)	33 (29.7)	26 (33.8)	7 (18.4)	17 (48.6)†	6 (42.9)†
Hypertension duration (years)	11(5–20)	10(3–16)	12(8–22)*	15(8–22)*	8.0(3.8–15.3)	20.1(10.4–29.2)†	15.0(5.8–17.3)
Anti-hypertensive treatment (N,%)	146(89.6)	56(88.9)	103(92.8)	65(84.4)	31(81.6)	32(91.4)	11(78.6)
Statin use (N,%)	6(3.7)	6(9.5)	5(4.5)	4(5.2)	3(7.9)	3(8.6)	1(7.0)
Body mass index (kg/m2)	24.8±3.7	25.3±3	25.1±2.8	25.3±2.9	24.8±2.5	25.4±3.4	25.1±2.8
Systolic blood pressure (mmHg)	136.3±16.5	139.4±15.3	142.6±16.6†	143.9±19.9	140.1±17.2	141±18.3	149.6±19.6†
Diastolic blood pressure (mmHg)	73±9.9	72.4±10.2	74.2±10.9	71.4±10.3	71.3±9.4	70.8±9.9	72±11.4
Heart rate (beats/min)	76.4±13.2	74.4±12.1	73.4±10.2*	74.6±10.4	74.8±11	70.5±10*	79±12.9
Plasma glucose (mmol/L)	5.1±1.4	4.8±0.7	5.1±1	5.5±1.4*	5±1.2	5.5±1.3	5.8±1.7
Total cholesterol (mmol/L)	4.8±0.8	5±0.8	4.8±0.9	5±0.8	5±0.8	4.8±0.7	5.4±0.6*
LDL (mmol/L)	2.9±0.7	3.1±0.7	2.9±0.8	3.2±0.8†	3.2±0.7*	3±0.8	3.5±0.6†
HDL (mmol/L)	1.2±0.3	1.2±0.2	1.2±0.3	1.1±0.2*	1.2±0.2	1.1±0.2†	1.1±0.1
Triglyceride (mmol/L)	1.6±0.8	1.6±0.8	1.7±0.9	1.7±0.8	1.6±0.8	1.9±0.8	1.4±0.7
Serum creatinine (mmol/L)	65.3±19.8	68.3±18.9	70.2±19.1*	69.5±20.3	69.2±16.7	72.7±20.0*	60.6±14.4
Serum urea nitrogen (mmol/L)	5.6±1.3	5.7±1.4	5.7±1.3	5.5±1.3	5.4±1.3	5.6±1.2	5.6±1.4
Uric acid (mmol/L)	321.3±71.8	332.5±70.8	335.2±73.9	333.4±75.5	325.8±73.5	346.5±77.3	309.6±67.6
ACR (mg/mmol)	3.1(1.8–6.0)	2.8(1.9–3.9)	3.8(2.0–6.7)	3.9(2.3–7.8)	3.0(1.8–5.3)	4.3(2.5–6.3)	3.9(2.9–10.3)
Neutrophil account (*109/L)	3.6(2.9–4.4)	3.7(3.0–4.5)	3.6(3.0–4.6)	3.9(3.3–4.7)*	4.1(3.5–4.0)	4.3(3.2–5.2)*	3.4(3.2–4.0)
Lp-PLA_2_ (ng/ml)	206.6(109.8–340.8)	217(118.4–408.8)	249.6(131.8–430.2)*	266.2(152.4–443.1)†	239.2(10.8.0–415.0)	266.2(154.8–461.6)*	309.3(266.4–536.9)†

Data are expressed as mean ± SD, median (interquartile range), or percentage (%). Each group was compared with the control group using χ2 or Student t test. ECAS, extracranial arterial stenosis; ICAS, intracranial arterial stenosis; COMB, combined extra- and intracranial arterial stenosis. LDL, low-density lipoprotein; HDL, high-density lipoprotein;ACR, urine albumin creatinine ratio Lp-PLA_2_, Lipoprotein-associated phospholipase A2. In the comparison among groups, hypertension duration, ACR and Lp-PLA_2_ are log-transformed. ICAS/ECAS absent group is considered as control.

* p<0.05

† p<0.01.

### Lp-PLA_2_ass and ICAS/ECAS

In models only adjusted with sex and age, the Lp-PLA_2_ mass was significantly associated with isolated ICAS (OR: 2.3; 95% CI: 1.14–4.64), and concurrent extraintracranial stenosis (OR: 3.93; 95% CI: 1.62–9.51) (Table 2). Considering that Lp-PLA_2_ mass was not related to isolated ECAS (OR: 1.54; 95% CI: 0.68–3.48), we further estimated the relationship of Lp-PLA_2_ mass and complex ICAS, which referred to ICAS no matter how the extracranial arteries was. A significant association was found between Lp-PLA_2_ mass and complex ICAS (OR: 2.92; 95% CI: 1.54–5.54). After additional adjustment for other risk factors, including BMI, hypertension duration, current smoking and drinking status, diabetes, LDL, HDL, plasma glucose, mean arterial pressure, heart rate, neutrophil account, urine albumin creatinine ratio, serum creatinine, anti-hypertensive treatment and statin use, the strength of association attenuated but was still significant.

**Table 2:** Associations of Lp-PLA_2_ mass with intracranial and extracranial arterial stenosis.

	Model I			Model II		
	OR	95% CI	P	OR	95% CI	P
Isolated ICAS	2.30	1.14–4.64	***0*.*020***	2.27	1.11–4.65	***0*.*025***
Isolated ECAS	1.54	0.68–3.48	0.301	1.3	0.5–3.35	0.589
COMB	3.93	1.62–9.51	***0*.*002***	3.73	1.24–11.25	***0*.*020***
Complex ICAS	2.92	1.54–5.54	***0*.*001***	2.97	1.36–6.48	***0*.*006***
Isolated moderate to severe ICAS	3.63	1.16–11.30	***0*.*026***	3.17	0.68–14.70	0.140
Isolated moderate to severe ECAS	1.17	0.44–3.14	0.757	0.96	0.30–3.12	0.950
moderate to severe COMB	15.39	2.03–116.55	***0*.*008***	13.41	1.72–104.42	***0*.*013***
Complex moderate to severe ICAS	3.57	1.37–9.33	***0*.*009***	3.53	1.32–9.46	***0*.*012***

OR, odds ratio. Model I adjusted for age and sex. Model II adjusted for age, sex, BMI, hypertension duration, current smoking and drinking status, diabetes, LDL, HDL, plasma glucose, mean arterial pressure, heart rate, neutrophil account, urine albumin creatinine ratio, serum creatinine, anti-hypertensive treatment, and statin use. The odds ratio expressed the risk in the ICAS and ECAS group compared with the non-stenosis group. Isolated ECAS, extracranial arterial stenosis only; Isolated ICAS, intracranial arterial stenosis only; COMB, combined extra- and intracranial arterial stenosis. Complex ICAS, intracranial arterial stenosis no matter how was the extracranial arteries; Hypertension duration, neutrophil account, serum creatinine, urine albumin creatinine ratio and Lp-PLA_2_ are log-transformed.

### Lp-PLA_2_ mass and severity/location of ICAS/ECAS

Similarly, in simple adjusted logistic regression analysis, Lp-PLA_2_ mass were significantly associated with isolated moderate to severe ICAS (OR: 3.63; 95% CI: 1.16–11.3), concurrent moderate to severe extraintracranial stenosis (OR: 15.39; 95% CI: 2.03–116.55), and complex moderate to severe ICAS (OR: 3.57; 95% CI: 1.37–9.33). However, after multivariate adjusted logistic analysis, only concurrent stenosis and complex ICAS showed association with Lp-PLA_2_ mass, which might result from the relative small amount of moderate to severe ICAS.

**Fig 1:**
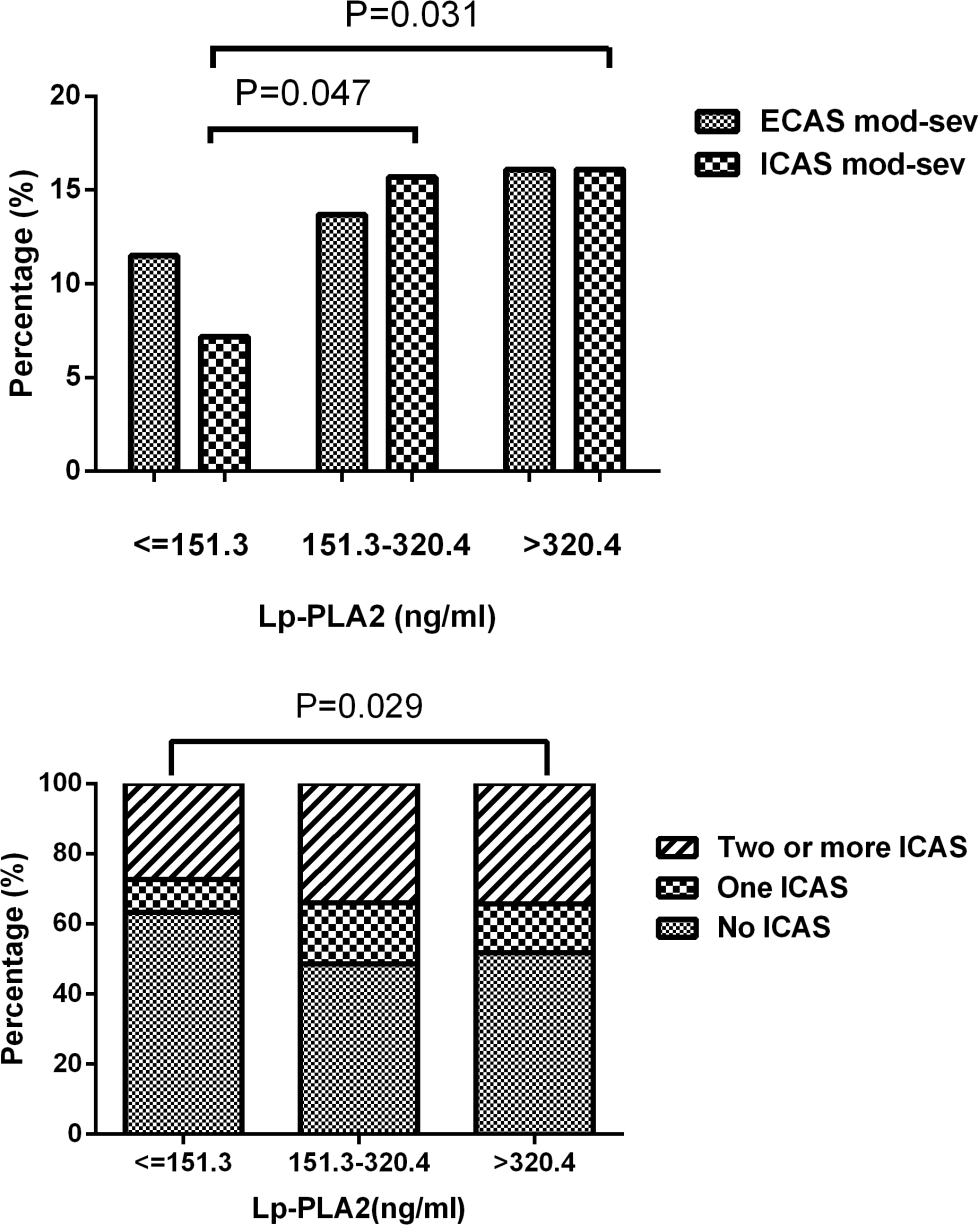
Prevalence of moderate to severe ECAS and ICAS (%) (A) and distribution of ICAS vessels (B) according to tertiles of Lp-PLA_2_. ECAS, extracranial arterial stenosis; ICAS, intracranial arterial stenosis; Lp-PLA2, Lipoprotein-associated phospholipase A_**2**_.

Compared to the subjects in the lowest tertile of Lp-PLA_2_ mass (11.5%), more subjects in tertile 2 (13.7%) and tertile 3 (16.1%) suffered moderate to severe ICAS (P < 0.05) (Fig 1A). Moreover, patients in the highest tertile of Lp-PLA_2_ mass showed higher frequency of multiple ICAS (34.3%) than the lowest tertile group (27.3%) (Fig 1B). The severity or number of ECAS was not related to the Lp-PLA_2_ mass level.

### Sensitivity test

We also checked the consistency of our results for complex ICAS according to various baseline characteristics (Table 3). After the subjects were subdivided according to median of age (65.8y), the magnitude of the association was similar among groups. The results for women and subjects with LDL >3.0 mmol/L were similar to the results of combined analyses of data. However, in men and subjects with LDL < = 3.0 mmol/L group, the association of Lp-PLA_2_ mass and ICAS was not significant.

**Table 3:** Associations of Lp-PLA_2_ mass with complex intracranial arterial stenosis in subgroup analysis.

	OR	95% CI	P
Men	0.99	0.64–6.18	0.233
Women	5.00	1.47–17.09	***0*.*010***
Age < = 65.8y	3.46	1.33–8.98	***0*.*011***
Age > 65.8y	2.42	1.01–5.81	***0*.*047***
LDL< = 3.0 mmol/L	2.47	0.84–7.26	0.100
LDL>3.0 mmol/L	5.29	1.43–19.5	***0*.*012***

## Discussion

In this stroke-free Chinese hypertension population who underwent both intra- and extracranial CTA, a significant and independent association was found of Lp-PLA_2_ mass with ICAS and concurrent stenosis. No significant difference of Lp-PLA_2_ mass was detected among patients with or without ECAS. Moreover, subjects with higher Lp-PLA_2_ mass showed more sever ICAS and had more intracranial arterial lesions.

As a minimally invasive imaging modality, CTA had been proved to provide better delineation of the anatomy of intra- and extracranial arteries [12,13], thus yielding higher diagnostic accuracy of the luminal stenosis of ICAS as compared with TCD and MRA, with DSA as the reference standard [14]^.^ In the present study, we evaluated the stenosis of both intra- and extracranial arteries by CTA, which could increase the sensitivity and accuracy of stenosis estimation.

Although the vascular risk factors were similar in the process of atherogenesis, the distribution and severity of atherogenesis varied among different individuals, whereas patients of Asian, African and Hispanic ancestry were at high risk of ICAS [15], while whites more frequently suffered from excranial carotid lesions [16]. Several previous studies had showed the different risk factors for ICAS and ECAS. In a study including 425 stroke-free Japanese patients, Uehara et al. found that the independent predictors of ECAS were age, hyperlipidemia and ischemic heart disease (IHD), while those for ICAS were age, hypertension, diabetes mellitus and IHD [17]. The study of Lopez-Cancio et al. in a Spanish population found that male sex, hypertension, smoking were independent risk factors of isolated ECAS, and diabetes and metabolic syndrome conferred a higher risk for ICAS [18]. Similar with the former studies, we found that the subjects with isolated ECAS were younger, and showed higher TC, lower SBP and shorter hypertension duration than those with isolated ICAS.

Since the first report in 2000 [19], a great deal of transectional and prospective studies had investigated the association between circulating Lp-PLA_2_ and ischemic stroke. In a population-based study, Oei et al. found the subjects with the fourth quartile of Lp-PLA2 activity were at the high risk for ischemic stroke [9]. The Atherosclerosis Risk in Communities Study underwent a case-control study and found that Lp-PLA_2_ and hs-CRP were useful intermediate risk factor for ischemic stroke in addition to traditional factor [20]. The meta-analysis performed by Lp-PLA_2_ studies collaboration, including 79036 participants in 32 prospective studies confirmed the association of Lp-PLA_2_ mass with ischemic stroke [21]. A study of symptomatic patient population also found Lp-PLA_2_ activity was increased in patients with multiple or bilateral stenosis [22].

We hereby reported a significant and independent association of Lp-PLA_2_ mass with ICAS, but not with ECAS. The reasons that explained the different effect of Lp-PLA_2_ on the distribution of atherosclerotic lesions were not well known. Even though it was the case that atherosclerosis was a systemic disease and the vascular system is uniformly exposed to risk factors, the inflammatory responds seemed differentially regulated in certain site-specific pattern. Mohler et al. had observed the lesions development of coronary arteries, thoracic aorta and carotid arteries in swine for nine months, in which they found expression of Lp-PLA_2_ was unregulated only in coronary but not carotid arteries [23]. Rotterdam Study also showed Lp-PLA_2_ was not associated with carotid arteries atherosclerosis [24], which was in accordance with our findings. Several theories provided cues for the site specific impact of risk factors on arteries, including different anatomical origins [25], various shear stress dependent endothelial gene expressions [26], and diverse shear stress dependent accumulation of inflammatory cells in specific vascular regions [27]. Furthermore, the additional risk factors might also influence the effect of Lp-PLA_2_ on extraintracranial stenosis. In this study, all the subjects had the history of hypertension, which probably act synergistically with Lp-PLA_2_ during the formation of ICAS.

In the sensitivity analysis, we found the association of ICAS and Lp-PLA_2_ only existed in women and patients with higher LDL. Researchers had suggested that the relationship between Lp-PLA_2_ and cardiovascular diseases might be influenced by the level of plasma lipid. In a study with 580 hyperlipidemic men, Lp-PLA_2_ was approved to be a strong predictor of coronary heart disease [19]. Contrarily, the study performed in healthy middle-aged women with relatively low plasma lipid achieved opposite result [28]. In our population, most of the women were over 60 years old, and displayed higher LDL than men. It might be the LDL level but not the gender which could impact the effect of Lp-PLA_2_ on ICAS.

Several limitations of our study should be considered. First, we measured Lp-PLA_2_ mass only, which might miss some information about Lp-PLA_2_ activity and ICAS or ECAS. But, it was reported that the correlation between Lp-PLA_2_ mass and Lp-PLA_2_ activity was about 0.5 [21]. Moreover, the Lp-PLA_2_ mass and Lp-PLA_2_ activity had similar predictive power for the cardiac death [29] and stroke [30]. The American Association of Clinical Endocrinologists’ (AACE) also recognized that Lp-PLA_2_ mass over 223ng/ml could be considered as a part of a global risk assessment strategy for patients with dyslipidemia and other major cardiovascular disease risk factors [31]. The Second, we didn’t measure the stability of atherosclerosis plaque in extraintracranial arteries, which had been considered as an important index for prediction of ischemia stroke, and might be related to the inflammatory procedure of Lp-PLA_2_. However, researchers had proved that ICAS rather than carotid plaque played more significant role in stroke incidence and recurrence in blacks, Hipanics and Asians [32]. Evidence from the Chinese population also indicated that carotid atherosclerosis was not independently correlated with ICAS [33]. The association of Lp-PLA_2_ with ICAS we found here might give some clues to understand the process from ICAS to stroke, especially in Asians. The third, patients under current study had a relative low cardiovascular risk profile and had few sever ICAS, which might therefore not be directly generalized to high risk patients.

In conclusion, this study revealed an association between Lp-PLA_2_ mass and ICAS, but not ECAS, in stroke-free hypertension patients of Chinese population. Patients with higher Lp-PLA_2_ mass showed more sever ICAS and had more intracranial arterial lesions. Studies based on Lp-PLA_2_ activity and other inflammatory measurements were suggested. In addition, the further long-term cohort study was warranted to elucidate the concrete effect of Lp-PLA_2_ on the asymptomatic ICAS.
